# Aptamer-based assays: strategies in the use of aptamers conjugated to magnetic micro- and nanobeads as recognition elements in food control

**DOI:** 10.1007/s00216-021-03501-6

**Published:** 2021-07-10

**Authors:** Monica Mattarozzi, Lorenzo Toma, Alessandro Bertucci, Marco Giannetto, Maria Careri

**Affiliations:** Department of Chemistry, Life Sciences and Environmental Sustainability, University of Parma, Parco Area delle Scienze 17/A, 43124 Parma, Italy

**Keywords:** Aptamer, Magnetic beads, Electrochemical aptasensor, Optical apta-assay, Magnetic solid-phase extraction, Food control

## Abstract

An outlook on the current status of different strategies for magnetic micro- and nanosized bead functionalization with aptamers as prominent bioreceptors is given with a focus on electrochemical and optical apta-assays, as well as on aptamer-modified magnetic bead–based miniaturized extraction techniques in food control. Critical aspects that affect interaction of aptamers with target molecules, as well as the possible side effects caused by aptamer interaction with other molecules due to non-specific binding, are discussed. Challenges concerning the real potential and limitations of aptamers as bioreceptors when facing analytical problems in food control are addressed.

**Graphical abstract:**

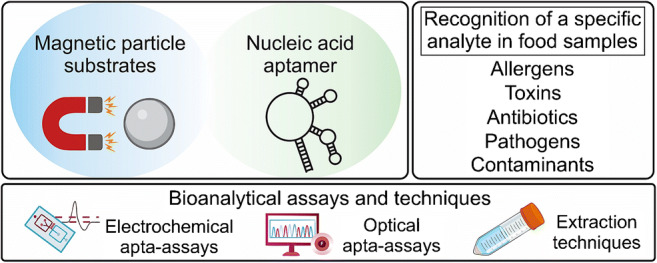

## Introduction

Aptamers, first developed in 1990 by three groups independently, are structured RNA or DNA motifs selected in vitro for the ability to bind with high affinity (K_d_ values in the low nanomolar to picomolar range for proteins and in the nanomolar to micromolar range for small molecules) and specificity to a wide range of target molecules including proteins or small molecules [1]. This property has created new opportunities for the design of sensors and other assays to address the continuing need for accurate analytical tools [1, 2].

Aptamers are emerging as attractive alternatives to antibodies because they offer significant advantages over them, such as easy, highly reproducible synthesis, convenient chemical modification with functional groups enabling custom tailored properties, higher stability, and consequently longer shelf life [3].

Aptamers show also certain advantages over other artificial recognition systems such as molecular imprinted polymers (MIPs). Despite their easy synthesis and wide applicability, MIPs imprinted with one type of target analyte, so-called template molecule, show high cross-reactivity, exhibiting recognition to related and sometimes also to apparently unrelated molecules [4]. On the other hand, aptamers suffer from limitations related to their rapid degradation in biological fluids due to interactions with biomolecules and to the dependence of their tertiary structure on solution conditions. Conformational flexibility is supposed to be an important factor limiting the affinity and specificity of interactions owing to the entropic cost associated to ligand binding [5]. Sometimes, cross-reactivity of aptamer-based assays towards a set of ligands may also occur, when aptamers bind to structurally similar non-target compounds causing unspecific binding and leading to unwanted side effects [6]. In order to address this issue, which is a drawback of the conventional in vitro systematic evolution of ligands by exponential enrichment (SELEX) selection method using nitrocellulose membrane [7], conditions of filter binding SELEX method for aptamer engineering need to be optimized. Among the possible different strategies, the introduction of a negative control selection step with structurally similar molecules into the SELEX method could result in minimizing cross-reactivity [8]. High-affinity aptamers can also be identified by enrichment rate and optimization of aptamer sequences to improve their binding properties and increase their affinity and specificity [9]. By using this approach, deep sequencing of reference targets has proved useful for eliminating candidates with non-specific binding.

Among different selection approaches for SELEX, techniques using magnetic beads as substrates for aptamer immobilization were developed to address some of the constraints of filter-based SELEX methods, including their high levels of background binding and their applicability limited to proteins. With the prospect of using SELEX technology to the generation of DNA ligands for small molecule targets, magnetic beads equipped with a linker arm for immobilization were used for the first time in 1997 [10]. Magnetic bead–based SELEX offers the advantage of allowing selections to be carried out against any target that can be immobilized onto the beads. In addition, aptamer selection processes can be carried out in smaller volumes with the smallest feasible amount of target, and magnetic particles can greatly facilitate the isolation process of the aptamer-coated bead–target complex simply by the application of a magnetic field.

These properties of microsized magnetic beads (MBs) also make them versatile tools for the isolation, detection, and determination of analytes from complex matrices, such as food and biological samples, by attaching aptamers on the surface of particles. Magnetic nanoparticles (MNPs) to be functionalized with aptamers have also been investigated [11]. However, binding of aptamer bioreceptors onto the surface of the magnetic particles requires an optimal functionalization of MBs using bioconjugation chemistries for developing MB-based assays [12]. In fact, previous investigations had shown that certain conjugation strategies resulted in a higher degree of nonspecific binding than others because of the particle modifications required for bioconjugation [13]. On this basis, in a study dealing with the evaluation of different approaches for immobilization of a DNA aptamer developed against the allergenic peanut protein Ara h1 on magnetic beads, three parameters were considered, i.e., particle surface coverage with aptamer bioreceptors, efficiency in capturing target protein by the functionalized beads, and non-specific protein adsorption on the functionalized particles with the ultimate goal of optimizing surface chemistry for coupling Ara h1 aptamer to MBs [12]. Non-specific interactions between the target allergenic protein and the particle surface were found to be dependent on the characteristics of the particle surface, i.e., hydrophilic, highly negatively charged surface or hydrophobic, low-charged surface, thus highlighting the important role of the selection of the optimal particles for bioassay development, in which non-specific interactions can occur in complex matrices as food.

In general, as reported in a review article, the potential free sites on the particle surface left after immobilization of the aptamer are still subject to binding oligonucleotides, which raises concerns of non-specific enrichment of the random library [14] and can cause artifacts if not properly considered in the development of apta-assay. Furthermore, a common limitation of the aptamer-based assays is the possible drastic changes in their binding properties due to their immobilization on solid substrates such as MBs or MNPs.

As MBs represent an attractive aptamer immobilization support in the SELEX process and for analytical applications, this feature article discusses different strategies in the use of aptamers conjugated to magnetic beads as recognition elements in food control, with a focus on electrochemical and optical apta-assays as well as on aptamer-modified MB-based miniaturized extraction techniques. Aptamer-immobilized MNPs are also discussed in the same field of analytical applications. In addition, the present work gives insight into critical aspects that strongly influence aptamer–target interactions, with the aim of improving awareness about the risk of result misinterpretation due to non-specific binding to immobilized aptamers or to the solid surface of magnetic particles. Finally, it is highlighted how more emphasis should be put on the investigation of matrix effects of magnetic particle–based apta-assays.

## Magnetic micro- and nanobead-based electrochemical aptasensors

Aptamer-based electrochemical biosensors have demonstrated great potential in analytical chemistry because of their simplicity, inherent miniaturization, and the possibility to achieve ultrasensitive quantification with low cost compared with other analytical strategies. In particular, the peculiar features of DNA/RNA-based aptamers and the versatility of their synthesis and functionalization allow their use as bio-recognition elements for the development of aptasensors with electrochemical transduction. For this purpose, aptamers are commonly 3′- and/or 5′- functionalized with electroactive or enzyme tags and with moieties suitable for their immobilization on electrodes or magnetic substrates. Electrochemical aptasensors can be classified on the basis of the signal-generating tag or according to the mechanism of interaction with the target, i.e., competitive or non-competitive. In the non-competitive mechanism, which is generally used for aptamers directly immobilized on electrode surfaces and involves the use of a single oligonucleotide probe, the interaction of the latter with the target analyte induces a conformational change that results in the tag being in proximity to or far away from the electrode surface.

The transduction can also be performed using a label-free approach, which does not require a tag on the immobilized aptamer, but uses a free redox probe mixed with the sample. Sample diffusion towards the electrode is influenced by the interaction of the aptamer with the target with a consequent conformational change. This approach is frequently associated with high background signals and non-specific binding, thus limiting its reliability and applicability to complex matrices.

The immobilization of aptamer probes on magnetic microbeads and nanobeads, bearing on their surface reactive functional groups such as carboxylic and amino units or streptavidin, provides several advantages since the interaction of the DNA/RNA probe with the target analyte occurs in suspension, exploiting an enhanced active surface under mechanical stirring and temperature control. It should be noted that these experimental parameters, which are crucial for an effective aptamer–target interaction, cannot be controlled when the aptasensor is based on direct immobilization of the probe on the electrode surface. Aptamer-functionalized magnetic materials suspended in the sample are subsequently isolated, washed, and transferred on screen-printed electrodes, free from any functionalization with receptors; all these operations are carried out using a simple magnet. Electrochemical aptasensors implemented on magnetic particles are usually based on a competitive mechanism for signal generation. A typical competitive setup involves the use of the oligonucleotide probe linked to the magnetic surface, and competitive reaction takes place between the target analyte and a labeled second probe having a nucleotide sequence partially complementary to the aptamer bound on the magnetic substrate; in the absence of analyte, the probe aptamer and its complementary sequence are hybridized, and the electroactive tag generates the signal (on), progressively decreasing as the target analyte concentration increases (signal-off). These phenomena take place since the binding of the target analyte to the immobilized probe induces the displacement of the labeled complementary sequence.

An alternative competitive mechanism involves the use of a single unlabeled aptamer immobilized on magnetic particles, while the target analyte present in the sample is put in competition with its labeled homologous at fixed concentration in order to generate a dose-response curve in which the electrochemical signal decreases as the concentration of the native analyte present in the sample increases. This approach is particularly suitable for the determination of proteins, which can be easily labeled with electroactive or enzymatic tags. This also allows for evaluating the degree of affinity between the immobilized probe and the analytes in solution, also through screening tests aimed at comparing the performance of different aptamers.

The ever-growing interest in monitoring health-threatening contaminants in all stages of food production process led to an increasing development of a variety of aptasensors for their detection, since the food sector demands fast, sensitive, and cost-effective analytical approaches to food safety testing [15]. Literature data report several examples of electrochemical aptasensors developed on magnetic microbeads or nanobeads mainly aimed at determining small molecules such as toxins, antibiotics, hormones, and pesticides for food safety applications. Recent review articles discuss important achievements in the development of MB-based electrochemical aptasensors for the detection of hazardous substances in food including allergenic or pathogenesis-related proteins [15–19]. In this context, a dual-aptamer-based sandwich assay was developed for electrochemical detection of *Staphylococcus aureus*; streptavidin-coated MBs were functionalized with a biotinylated anti-*S. aureus* aptamer and suspended in the sample in order to capture the bacterium through specific recognition of its specifically expressed protein [20]. The second anti-*S. aureus* aptamer was immobilized on silver nanoparticles, thus obtaining a sandwich structure. Acid dissolution of the sandwich yielded silver ions, whose quantification was carried out by differential pulse stripping voltammetry. However, it should be noted that the experimental conditions for binding of aptamers immobilized on MB and silver nanoparticles to the pathogenic protein target, such as buffer solution, ionic strength, etc., are not reported and discussed by the authors with reference to the analysis of real water samples such as tap water.

As for the determination of allergenic proteins, a competitive approach based on the use of two aptamers binding 33-mer gliadin peptide was developed for sensitive gluten analysis in gluten-free food products [21]. For this purpose, the target peptide was immobilized onto streptavidin-coated MBs in combination with a low amount of biotin-labeled aptamer, followed by streptavidin-peroxidase labeling of the aptamer bound to the magnetic particles. The electrochemical signal associated with the enzymatic activity was finally read by chronoamperometry on screen-printed carbon electrodes (SPCEs). A noteworthy result of this study is the fact that the assay relying on the use of the aptamer with the highest protein binding affinity, i.e., Gli-4, exhibited excellent analytical performance when analyzing samples containing gliadin as a non-hydrolyzed protein, whereas it fails to detect the target peptide in solution. Even though this drawback was faced by using the other studied aptamer (Gli-1), the most abundant one in the SELEX pool, which shows good performance for the determination of the allergen in hydrolyzed food samples, these findings demonstrate that specificity of aptamers towards target molecules is affected by sample composition including interfering components. In addition, different protocols were developed for gliadin extraction from unprocessed raw materials and processed food samples, using, respectively, a 60% ethanol solution and a cocktail solution containing denaturing agents, which are conditions normally required for a proper gliadin recovery.

**Fig. 1:**
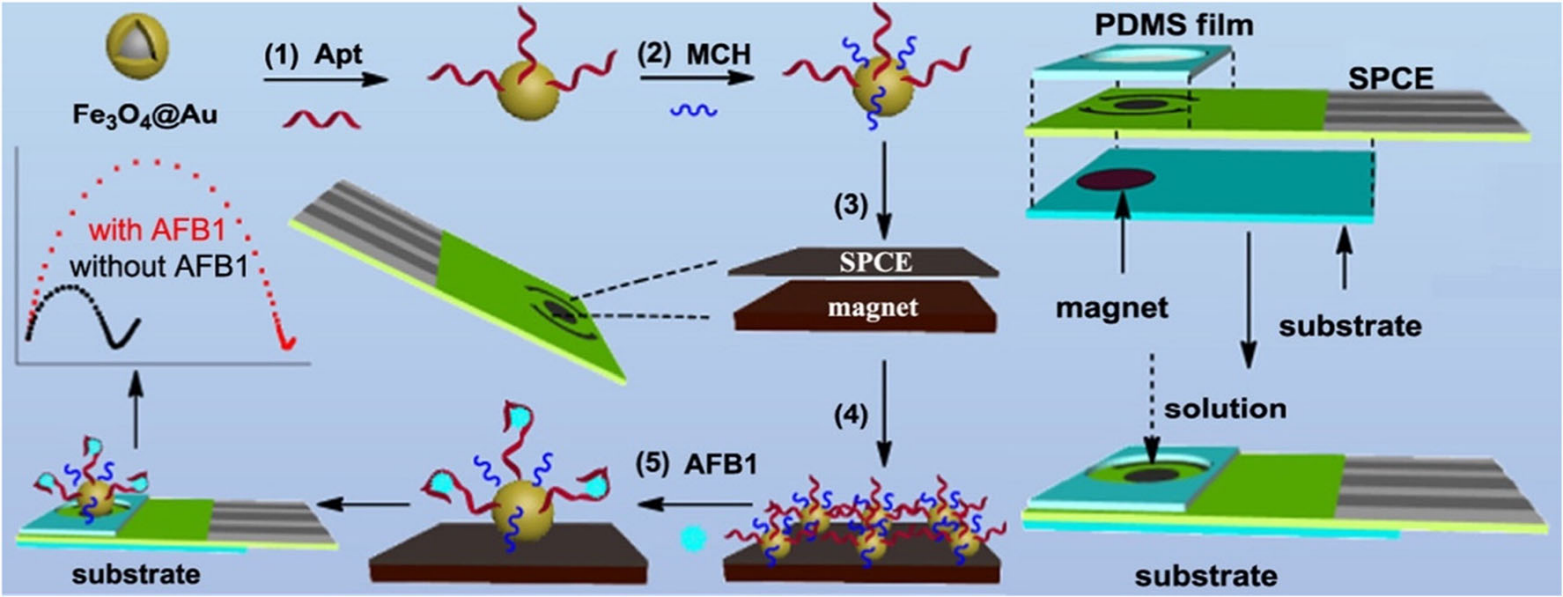
Schematic illustration of the fabrication of the magnetically assembled aptasensor for label-free determination of aflatoxin B1 exploiting electrochemical impedance spectroscopy (EIS) and the aptasensing device. Apt = aptamer, MCH = 6-mercapto−1-hexanol, PDMS = polydimethylsiloxane, SPCE = screen-printed carbon electrode, AFB1 = aflatoxin B1. Adapted from [22] Copyright 2018, with permission from Elsevier

Concerning other frequently encountered food contaminants, a magnetic aptasensor for label-free determination of aflatoxin B1 (AFB1) exploiting electrochemical impedance spectroscopy as transduction system was recently devised [22]; a 50-mer DNA aptamer probe bearing a thiol function at the 3′ was firstly immobilized on Fe_3_O_4_@Au nanospheres and magnetically transferred on disposable SPCEs covered with a polydimethylsiloxane film after incubation of AFB1 (Fig. 1). The electron transfer resistance progressively increased when the sensing interface was incubated with increasing concentrations of aflatoxin, since the formation of the aptamer–AFB1 complex changed the structure of the aptamer from random coils to a G-quadruplex structure. Although the specificity of the selected 50-mer DNA aptamer was not proven using random oligonucleotide probes, the impedimetric aptasensor showed a good selectivity towards aflatoxin B2, ochratoxin, and fumonisin B1. The biorecognition event taking place on aptamer-Fe_3_O_4_@Au nanospheres was proven also in peanut samples spiked with AFB1. A recent interesting example of multiclass contaminant determination is focused on quantification of kanamycin, aflatoxin M1, and 17β-estradiol in milk by a simultaneously responsive microfluidic chip aptasensor based on magnetic tripartite DNA assembly nanostructure probes [23]; a two-duplex tripartite DNA aptamer nanostructure was assembled on the surface of MNPs for recognition of the target and its subsequent release in the supernatant phase. It is worth pointing out that the ultra-high sensitivity of the microfluidic aptasensor required rolling circle amplification (RCA) followed by magnetic separation and hybridization with three different lengths of complementary strands to the RCA products.

MB-based electrochemical aptasensors also find effective application for determination of antibiotics in food. Ampicillin was detected and quantified in milk with good recovery rates using a micromagnetic competitive aptasensor that also exploits the enhancing properties of multi-wall carbon nanotubes [24]. For this purpose, ampicillin-coated MBs were used to compete with the sample for biotinylated aptamers and to recognize a streptavidin–horseradish peroxidase conjugate. Simple and low-cost SPCE were suited for subsequent electrochemical signal readout, allowing to quantify ampicillin over a 10^−13^–10^−8^ mol/L linear range.

**Fig. 2:**
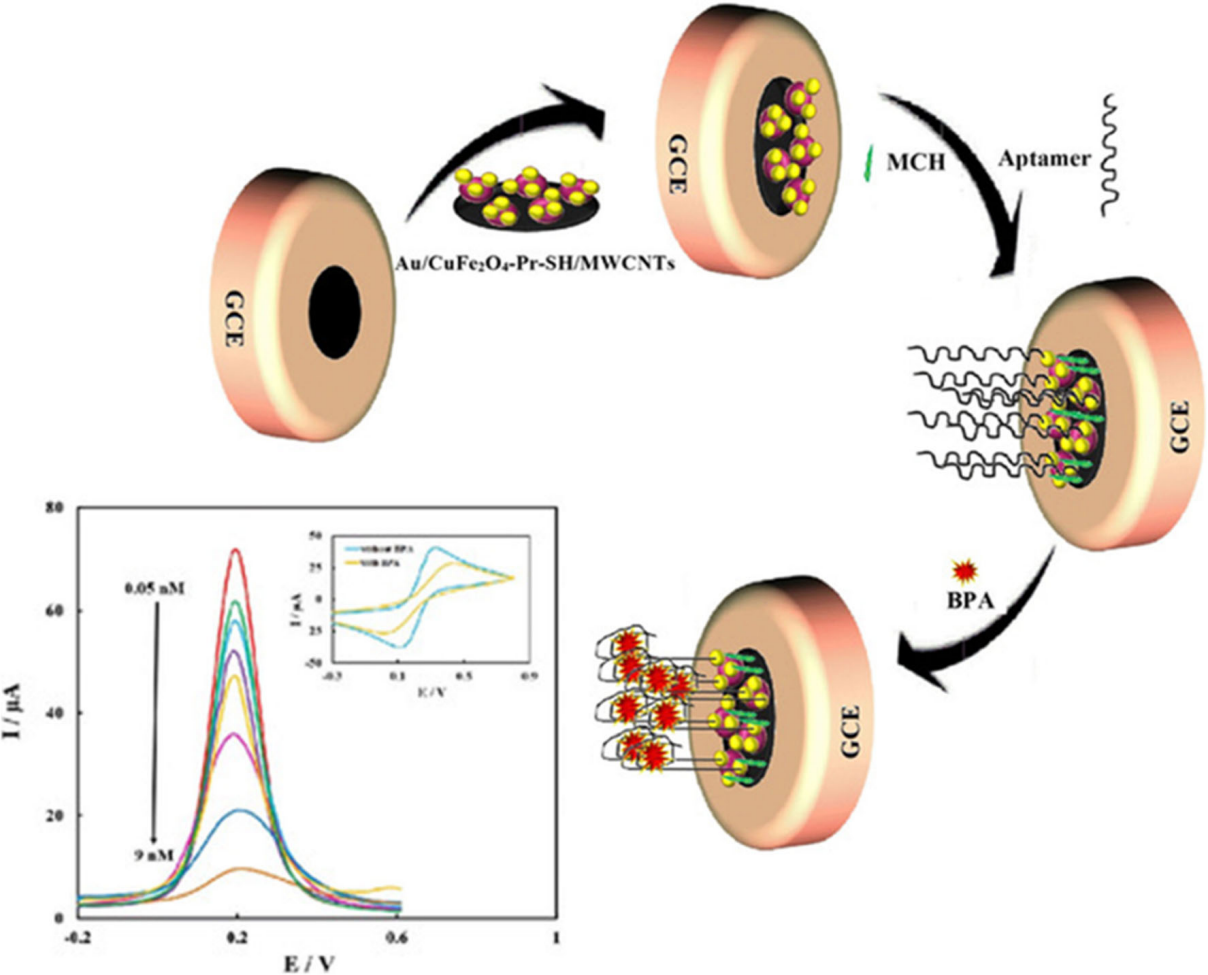
Working principle of a label-free electrochemical aptasensor for determination of bisphenol A based on magnetic gold nanocomposite. GCE = glassy carbon electrode, Pr-SH = (3-thiopropyl) triethoxysilane, MWCNTs = multiwall carbon nanotubes, MCH = 6-mercapto−1-hexanol, BPA = bisphenol A. Adapted from [25] Copyright 2018, with permission from John Wiley and Sons

As for application of magnetic gold nanocomposite-based aptasensors to the determination of endocrine disruptors like bisphenol A (BPA) in food, a label-free approach was exploited to develop an electrochemical aptasensor based on magnetic gold nanocomposite [25]. The aptamer chains immobilized on the surface of Au/CuFe_2_O_4_-Pr-SH/MWCNT nanocomposite modified SPCE act as long tunnels for the electron transfer mediated by [Fe(CN)_6_]^3−/4−^ redox probe (Fig. 2). In the absence of BPA target, the aptamer strands remain unfolded acting as an open gate for the electron transfer, while the binding of BPA induces a conformation change to a G-quadruplex structure, thus hindering the electron transfer and generating a signal-off current response. The magnetic competitive aptasensor was successfully applied to the determination of BPA in mineral water, milk, and juice samples.

The analytical characteristics of magnetic micro- and nanobead-based electrochemical aptasensors for food analysis selected from those discussed are illustrated in Table 1.

**Table 1:** Selected applications of electrochemical aptasensors and optical apta-assays based on magnetic materials for food analysis

Analytical approach	Target analyte	Matrix	LOD	Dynamic range	Reference
Electrochemical					
Streptavidin-coated MBs in combination with biotin–aptamer for electrochemical competitive assay	Gliadin	Gluten-free foods	4.9 μg/L	Not declared	[21]
Label-free impedentiometric magneto-assay based on thiolated aptamer-functionalized Fe_3_O_4_@Au nanospheres	Aflatoxin B1	Spiked peanuts	0.015 ng/mL	0.02–50 ng/mL	[22]
Competitive MB-based voltammetric aptasensor doped with multi-wall carbon nanotubes	Ampicillin	Milk	1.0 × 10^−13^ mol/L	1.0 × 10^−13^–1.0 × 10^−8^ mol/L	[24]
Label-free voltammetric aptasensor based on gold nanoparticles immobilized on copper MNPs and multi-wall carbon nanotubes	Bisphenol A	Milk, fruit juice	2.5 × 10^−11^ mol/L	5 × 10^−11^–9 × 10^−9^ mol/L	[25]
Optical					
Competitive fluorescence apta-assay based on recombinase polymerase amplification of protein-bound aptamer	β-Conglutin	Lupin	3.5 × 10^−11^ mol/L	Not declared	[27]
Fluorescent DNA-scaffolded silver nanocluster-based aptasensor	Ochratoxin A Aflatoxin B_1_	Wheat, rice, corn	0.2 pg/mL 0.3 pg/mL	0.001–0.05 ng/mL	[28]
Fluorescent competitive assay based on the formation of T-Hg^2+^-T base pairs	Mercury(II)	Ribbon fish	2.0 × 10^−10^ mol/L	2 × 10^−9^–16 × 10^−8^ mol/L	[29]
Multiplex fluorescence assay using multicolor upconversion nanoparticles as aptamer tags	*Staphylococcus aureus* *Vibrio parahaemolyticus* *Salmonella typhimurium*	Milk, shrimp	25 cfu/mL 10 cfu/mL 15 cfu/mL	5 × 10^1^–1 × 10^6^ cfu/mL	[31]

It has to be pointed out that a common shortcoming of the studies dealing with electrochemical aptasensors is the lack of a rational and exhaustive evaluation of the effects of experimental parameters such as ionic strength, pH, and temperature of the incubation medium of the target analyte, especially on matrix extracts. It is in fact well known how these factors drastically influence the binding properties of aptamer receptors, directly driving their conformation. Furthermore, when developing an aptasensor, the specificity of the interactions between the aptamer and the target analyte is rarely studied through appropriate experiments performed with reference probes, such as sequences randomized or unrelated to those selected with the SELEX methodology. Such studies, when carried out, often lead to surprising results questioning the properties of these latest-generation biomimetic receptors.

## Magnetic micro- and nanobead-based optical apta-assays

The use of aptamers conjugated to or in combination with magnetic beads and magnetic nanomaterials has also proved particularly useful in the design of analytical methods and molecular assays for food control with optical readout. Fluorescence spectroscopy is a commonly used technique in optical aptasensing methods, mainly due to its easy operation, its high sensitivity, and its great versatility given by the wide range of available fluorophores and nanomaterials that can be used as molecular tags or reporter systems. In this context, nanomaterials such as quantum dots and gold nanoparticles are especially useful in the development of Förster resonance energy transfer (FRET)–based sensing strategies because they provide highly efficient quenching mechanisms and chemical stability [26]. Optical measurements can also be performed based on colorimetric or chemiluminescence methods, which have the advantages of being rapid, relatively cheap, and simple to run. However, the general lower sensitivity of colorimetric techniques and the need for additional reactants or labeling in the case of chemiluminescence methods have put limitations on a more widespread utilization [26].

The high-affinity interaction between a specific analyte and its cognate aptamer in conjunction with magnetic particles is the key step underlying dynamic binding processes or triggering molecular reactions that can then be monitored via the selected optical technique. A common strategy of magnetic-based apta-assays in which the optical measurement is carried out in the supernatant following binding of the analyte to aptamer-modified particles and magnetic separation, is to leverage functional DNA strand exchange reactions that lead to the release into the supernatant of species enabling fluorescence-based detection, including quantum dots and optically active DNA sequences. It is also possible to design competitive apta-assays by following this approach. Binding of the target analyte to its cognate aptamer can prevent further association of the latter with a labeled complementary DNA sequence, which is then detected in the supernatant. Competitive colorimetric assays can otherwise be engineered that use an enzyme-labeled aptamer in the presence of both its target in solution, i.e., the analyte, and copies of the same target, i.e., the competitor, displayed on the surface of magnetic substrates. Alternatively, apta-assay can be designed in which the optical measurement is carried out on a fortified sample obtained from resuspension of analyte-enriched aptamer-modified magnetic particles. This strategy is ideal when the analyte displays multiple ligands available for interacting with their cognate aptamers, which is for instance typical of bacterial pathogens. Competitive apta-assays can also be engineered in which the fraction of the aptamer molecules bound to a target competitor displayed on the surface of MBs is amplified through nucleic acid amplification techniques and then detected by means of a specific fluorescence-based method [27]. This versatility in design has favored the development of a great variety of optical aptamer-based assays and technologies for the determination and quantification of specific food safety targets, including toxins, antibiotics, heavy metals, bacterial pathogens, and allergens [18, 19, 28–33].

Several studies related to the optical and electrochemical aptasensing of tetracyclines in food analysis were discussed in a recent review [19]. Fast and on-field apta-assay of antibiotics in real samples is highlighted, but also limitations resulting from the difficulty in bioconjugation to nanomaterials are discussed that may affect the efficiency of the aptasensors. New high-performance nanomaterials, as well as a deeper evaluation of the sample conditions and the nonspecific interactions of the aptamer with the sample matrix, can help overcome these issues.

**Fig. 3:**
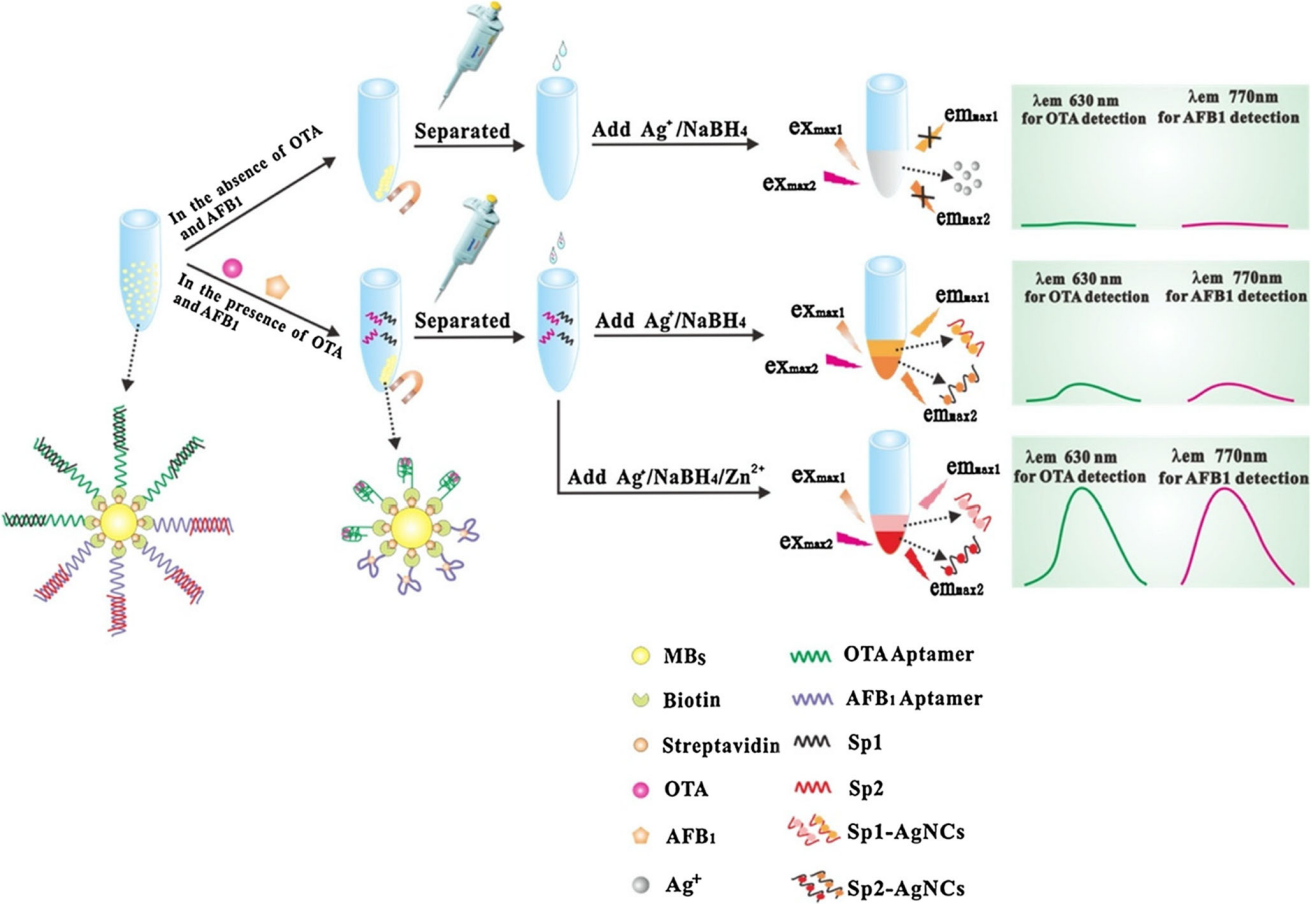
Principle of an optical assay for the simultaneous orthogonal detection of ochratoxin A and aflatoxin B1 based on the use of two specific aptamers and the formation of DNA-scaffolded fluorescent silver nanoclusters. MBs = magnetic beads, Sp1 = signal probe 1, Sp2 = signal probe 2, Sp1-AgNCs/Sp2-AgNCs = DNA-scaffolded silver nanoclusters using either Sp1 or Sp2, OTA = ochratoxin A, AFB1 = aflatoxin B1. Reprinted from [28] Copyright 2016, with permission from Elsevier

Food contaminants such as mycotoxins are especially dangerous because they can easily enter the food chain owing to their resistance to baking processes and can cause severe harm to consumers as the result of nephro-, hepato-, or neurotoxicity. Zhang et al. developed a fluorescent aptasensor for the simultaneous detection of ochratoxin A and aflatoxin B_1_ by combining the use of a pair of specific aptamers immobilized on the surface of MBs and signaling DNA probes (Fig. 3). The analyte-dependent formation of DNA-scaffolded silver nanoclusters enabled trace quantification of the two mycotoxins in cereal samples with LODs in the low parts-per-trillion level [28]. Determining possible contamination from mercury (II) ions is also critical in assessing the safety of edible fish. Sun et al. developed a mercury-binding aptamer to develop a highly specific, magnetic bead–assisted, competitive fluorescence assay for mercury detection and demonstrated its use in spiked fish samples [29].

Aptamers in association with magnetic materials are also powerful tools for engineering optical assays against bacterial pathogens in food samples. Infections from foodborne pathogen bacteria are a leading cause of many severe diseases and death worldwide; therefore, their accurate, sensitive, and rapid detection has taken a central role in food control. Conventional culture-based methods are cumbersome, time-consuming, and susceptible to contaminations. Apta-assays are a powerful alternative because they enable quantification of bacterial pathogens in food samples harnessing the specific binding between a selected aptamer and its target bacterium, which can be coupled with a wide range of strategies to produce an optical output [30]. Target bacteria can be specifically recognized by selected aptamers decorating the surface of magnetic materials. The resulting distribution of particles on the bacterial membrane allows the collection, extraction, or accumulation of the pathogens, which can be coupled with a wide range of detection strategies. However, as discussed in a recent review about optical aptamer-based assays harnessing magnetic beads for the detection of foodborne pathogenic bacteria, the size of the magnetic substrates used is a critical parameter. Micrometer-sized particles are easy to be recollected, but they may suffer from lower binding capacity and efficiency because of their reduced surface to volume ratio; conversely, nanometer-sized beads can provide a higher available surface area and a more homogeneous distribution of grafted aptamers, which can help improve the overall analytical performances of a designed apta-assay [30]. Wu et al. developed a multiplex apta-assay for the detection of *Staphylococcus aureus*, *Vibrio parahaemolyticus*, and *Salmonella typhimurium* by using three different specific aptamers labeled with fluorescent upconversion nanoparticles that are detached from MNPs in the presence of the target bacterium [31]. A possible drawback of these strategies lies in the high variability of the bacterial structure at the molecular level, which may affect the ability of an aptamer to recognize and bind to its target and thus the overall performance of an apta-assay.

Analytical strategies based on aptamers and magnetic particles play a strategic role also in effective monitoring of food allergy. In this context, optical apta-assays were developed which enabled detection and quantification of specific protein allergens, including β-conglutin, gliadin, Ara h1, and lysozyme, in food samples [32].

This wide range of applications demonstrates how aptamers used in conjunction with MBs or MNPs can be valuable tools for the engineering of optical detection strategies targeted to specific analytes in food control (Table 1). Recognition of a target ligand by an aptamer is the key molecular event that determines the specificity of the proposed method. In a representative study, Arshavsky-Graham et al. compared the ability of an aptamer and an antibody to bind properly to the same target protein when immobilized on a silicon surface used as an optical transducer [33]. They showed that the molecular orientation upon immobilization plays an important role in determining the resulting affinity of the bioreceptor. Aptamers are advantaged over antibodies because of their reproducible conjugation at the sequence terminus, which translates in improved sensitivity and specificity. However, a study by Amaya-Gonzalez et al. suggests that the performance of aptamer-based optical sensors may be influenced by variations in the affinity (dissociation constant) of the selected aptamer receptor for its target ligand resulting from the type and the position in the sequence of modifications such as biotin, fluorophore tags, and reactive functional groups that are necessary for surface anchoring or optical signaling [34]. The specific molecular conformation of a target in the sample, as well as the presence of interferents that induce alterations in the aptamer folding or in the structure of the target ligand, can also induce unexpected deviations in binding affinity and thus influence the overall efficiency of an apta-assay, as it was previously discussed in the case of electrochemical-based apta-assays [21]. It should also be pointed out that many of the strategies illustrated above are multistep as they are based on combinations of several molecular processes, including enzymatic amplifications, DNA-based reactions, and sequential recognition-binding events. Variations in the efficiency of any of these processes, as well as uncontrolled non-specific interactions, could end up adversely affecting the overall specificity and reproducibility of the methods. Sample pretreatment can also influence the analytical performance of these methods. Depending on the extraction procedure applied to food samples, the specific interaction between an aptamer and its target can be hindered by the presence of chemical or biological interferences that result in a reduction in selectivity and sensitivity.

## Aptamer-modified magnetic micro- and nanobead-based miniaturized extraction techniques

Analytical methods for food quality and safety assessment require adequate sample treatment to face the determination of analytes in complex matrices at trace levels. Consequently, a sample treatment protocol should provide efficient pre-concentration and enrichment of the compounds of interest to reach adequate sensitivity and selectivity, while minimizing matrix effect. Magnetic solid-phase extraction (MSPE) technique exploits MBs or MNPs as a miniaturized support for the sorbent, offering the advantages of increased contact surface with high adsorption/absorption efficiency, user-friendly operations, and versatility of experimental conditions. MSPE is normally operated by dispersing magnetic sorbent materials into the analyte-containing sample solution and recovering them with external magnetic field. The analytes are desorbed with small volumes of organic solvents from the sorbent. Washing cycles are required to remove non-specifically adsorbed/absorbed species, and finally, the retained compounds are properly eluted from the support for the subsequent instrumental analysis.

In the last years, efforts have been paid to couple miniaturized solid-phase extraction techniques as MSPE with molecular biorecognition elements using immunosorbents and oligosorbents [35], exploiting their affinity and selectivity/specificity towards target compounds.

**Fig. 4:**
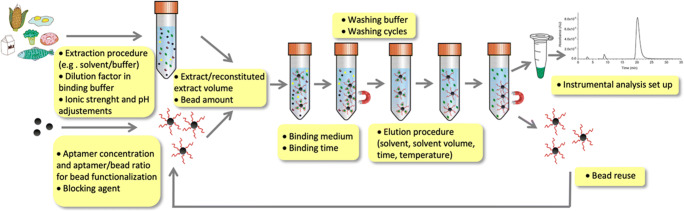
Graphical representation of the MSPE protocol based on aptamer-modified magnetic beads. The most critical analytical aspects in each step are highlighted

As shown in Fig. 4, many parameters involved in the enrichment and elution steps need to be optimized towards the development of an efficient MSPE procedure. When using aptamers as biomimetic receptors, the most critical factors are the composition, pH, and ionic strength of the binding medium since it strongly affects the structures of the aptamers as well as the binding process. The strict control of binding conditions is a critical aspect when moving from standard solutions in binding buffer to matrix extracts, where interfering compounds are also present. In this case, it is fundamental to find a compromise between binding conditions, able to maintain aptamer affinity and selectivity, and extraction buffer/solvent for a good recovery from food samples. In addition, in the case of proteins as target molecules, extraction conditions should preserve protein structure, avoiding denaturant or chaotropic agents that are often exploited to favor the extraction from processed foods, as in the case of allergenic proteins. However, it should be noted that this issue is still unexplored in the literature.

Despite the large number of aptamers selected for different targets, their reliable applications in MSPE are still in their infancy as well as limited to few small molecules. As for food safety and quality monitoring, published articles focus exclusively on mycotoxins or antibiotic residues [36–41].

An MSPE procedure was developed for the determination of aflatoxin B1 and B2 in maize by HPLC with fluorescence detection [36]. The extraction and reconstitution steps involved a methanol/water mixture in the presence of high concentration of NaCl, representing conditions not compatible with the aptamer–target interaction. Consequently, a proper dilution factor was studied to be applied to the reconstituted extract to reach the maximum concentration of methanol that would not hinder interactions. In addition, analyte recovery was found to decrease as the sample volume increased, proving that the sample volume during capture also affects the binding efficiency of the aptamers. Khodadadi et al. developed an aptamer-based MSPE method for HPLC–UV–vis determination of aflatoxin M1 in milk, performing a defatting step with hexane and extraction with methanol/water mixture (8:1), containing high NaCl concentration, then adding the functionalized MNPs directly in the extract filtrate [37].

In a recently published work dealing with the development of an aptamer-based MSPE followed by liquid chromatography–tandem mass spectrometry (HPLC–MS/MS) analysis for the determination of chloramphenicol residues, selectivity assessment towards various analogues and method validation were performed in binding buffer. The devised method was then applied to different food matrices, i.e., fish, chicken, and milk powder, and to serum: the extraction protocol was the same for all sample types and a final reconstitution volume was not declared. Surprisingly, the method performance does not appear to be compromised by the presence of interfering compounds for any of the matrices investigated, yielding recoveries in the 87–107% range at low parts-per-trillion (ppt) levels [38]. As for antibiotic residue determination in food, Huang at al. exploited the immobilization of an aptamer selective to three different relevant amphenicols on MNPs, followed by HPLC–UV instrumental analysis. The applicability of the devised protocol was tested in milk, diluting the matrix in LC mobile phase (methanol:water) before the MSPE enrichment step [39].

Guo et al. developed an MSPE procedure using aptamer-functionalized magnetic Fe_3_O_4_/graphene oxide/aptamer composite for the determination of sulfadimethoxine in milk; parameters such as extraction time, sample pH, amount of adsorbent, elution solvent, and elution time were studied in binding buffer [40]. The selectivity of the method was investigated by comparing the HPLC–UV chromatogram of a standard solution of four sulfanilamide antibiotics after MPSE procedure with that acquired before MSPE: the only signal showing a significant increase was that of the target analyte, whereas the peak areas of other compounds remained unchanged or one of them was not detectable. These results highlight selectivity allowing the enrichment of the target analyte by MSPE, but without binding specificity. In this regard, it would have been very interesting to also investigate the behavior of non-functionalized or functionalized magnetic adsorbents with scrambled oligonucleotide sequences. Aptamer-linked MNPs were exploited for the development of an automated enrichment approach for sulfanilamide in milk [41]; firstly, the selection of aptamer sequence towards the target analyte was carried out in a milk-simulating buffer having a pH value and ion conditions comparable to those of bovine milk. Despite a poor aptamer selectivity, especially towards chlortetracycline, manual and automated aptamer-based enrichment procedures were investigated in five matrices with different fat contents, i.e., milk-simulating buffer, skimmed milk, skim milk, full-cream milk, and cocoa milk drink, followed by spectrophotometric analysis. Good enrichment factors up to eightfold were obtained, with the automated process giving the highest reproducibility by reduced hands-on time.

## Open questions and challenges to be addressed

Due to their high sensitivity, low cost, and easy fabrication of sensing devices, fully integrated aptasensors and apta-assays provide real-time methods to detect the presence of contaminants, pathogenic bacteria, and allergenic ingredients in food matrices. However, challenges remain for the practical and reliable application of these bioassays which must be overcome. When working with complex samples such as food samples, the sensitivity and selectivity of apta-assays could be affected by the presence of interfering components (i.e., lipids, carbohydrates, proteins), pH, and ionic strength. In this regard, it is highlighted that in published articles there is often a lack of details and critical discussion about crucial aspects, namely how optimal conditions of the binding buffer can be reproduced for a matrix extract, which could contain organic solvents or denaturant agents used for analyte extraction. Even in the simplest case of tap water analysis, this aspect is not trivial: if the selective/specific binding event requires certain conditions, proper ionic strength or pH adjustments are strictly required. Too often, the applicability of a developed method to real samples and its performance seem predictable only on the basis of the efficiency of the aptamer as a bioreceptor for a certain target in the binding buffer. In addition, non-specific binding events could arise from a not effective or even absent blocking on the support surface, as well as from non-specific binding of aptamer to sample components that can lead to false-positive results.

Beyond the development of innovative aptamer-based sample treatment protocols, the implementation of apta-assays on magnetic beads could be a strategy to provide evidence of the aptamer binding to the target. In this case, an instrumental analysis downstream of the assay and able to monitor a signal uniquely ascribable to the target compound offers a second level of selectivity towards the target unlike electrochemical or optical transduction. In this way, it is possible to validate specific aptamer–target interactions and to study possible cases of non-specific binding. Things get even more complicated moving from buffers to matrix extracts, which represent the “real scenario” for analytical chemists. In this context, the possibility of reading the apta-assay with a mass spectrometry–based technique, as gas chromatography (GC)–MS or HPLC–MS, would allow not only the assessment of the selectivity towards a few interfering compounds in the binding buffer, but also the target or untarget identification of all compounds that have interacted with the aptamer-modified beads, since they are all present in the eluted phase.

As recently argued by Wu et al. [42], there is an urgent need to go beyond the “proof of concept,” exploring the real potentialities and limitations of aptamers as bioreceptors when facing analytical problems. Although thousands of papers have been published on aptamers, a rigorous multifaceted characterization of aptamer–target structures and the binding mechanism is still lacking, which would be critical for assessing the reliability of aptamers as bioreceptors [42, 43]. The scientific community is urgently called to fill the gap between aptamer selection and biosensing strategies, since unfortunately the risk to misinterpret the real performance of an aptamer sequence is high. In many cases, aptamer binding assays are not straightforward and can be prone to artifacts [19]. In this regard, it should be noted that two very recent studies disproved that some oligonucleotide sequences, widely used in literature for the development of aptasensors, do not actually bind to their expected target [43, 44]. A multi-technique platform used to characterize the aptamer–target interaction allowed demonstrating that arsenic-binding aptamer, previously used in more than two dozen papers, did not provide a specific binding of As(III), and the same binding trends were observed with a random DNA [44]. In 2020, Bottari and coworkers disproved the binding capability of aptamer for ampicillin, which was used as a biorecognition element in several detection strategies; the authors claim that caution towards the use of aptamers in each new application has considerably diminished over time [43], probably because the aptamer–target interaction is simply presumed without corroboration by independent evidence. In fact, in many cases, simple knowledge of the aptamer sequence seems to be enough: actually, if analytical researchers do not critically investigate all the conditions that may affect the aptamer binding process, considering also interfering compounds and control random sequences, the experiments may be meaningless [45]. Finally, cost-effective truncation of the aptamer sequence must be done in a rational way, since the truncated aptamer could provide a different performance than the original full-length oligonucleotide sequence [45].

## Outlook

Challenges remain for the practical application of aptamer-based assays which must be overcome. Therefore, further studies are required for the development of analytical methods to solve these problems and pave the way for practical application of these bioassays. In order to devise robust and reliable analytical strategies based on aptamer recognition, in our opinion, the following fundamentally important aspects need to be considered:

(i) Further research should be devoted to validating binding affinity and selectivity by means of a multi-technique approach, such as spectrophotometric, mass spectrometry–based, electrochemical and calorimetric characterization. This multi-platform assessment could also be useful to rationally face the probe truncation with respect to the full-length originary oligonucleotide sequence.
(ii) The suitability of the aptamer-based methodologies for food control issues requires an exhaustive evaluation of the effects ascribable to the properties of the matrix extract, i.e., pH, ionic strength, and matrix components, on the aptamer–target interaction.
(iii) The capabilities and limitations of the developed methods need to be further explored by performing negative controls involving the use of unrelated and scrambled aptamer sequences. Furthermore, a proper blocking stage must be carried out to avoid aspecific binding to the magnetic support.
